# Comparative Analysis of Extraction Complications in Transvenous Versus Leadless Pacemakers

**DOI:** 10.7759/cureus.108392

**Published:** 2026-05-06

**Authors:** Abdullah Sahyouni, Antonella Jimenez, Johny Alrahal, Andrew Knight, Roy Chen, Tanmayee Maddineni, Luis Antonio Lugo, Asmitha Koneru, Kurt Ramey, Robert J Subbiondo

**Affiliations:** 1 Internal Medicine, Blake Medical Center, Bradenton, USA; 2 Internal Medicine, University of South Florida, Tampa, USA; 3 Cardiology, HCA Florida Blake Hospital, Bradenton, USA; 4 Cardiology, Blake Medical Center, Bradenton, USA; 5 Cardiology, University of South Florida Morsani College of Medicine, Bradenton, USA

**Keywords:** cardiac implantable electronic device (cied), cardiac pacemakers, cardiac rhythm devices, cardiology devices, internal medicine-cardiology, life threatening arrhythmia, pacemaker extraction, pacemaker lead displacement, permanent pacemaker implantation (ppm), ppm complication

## Abstract

Pacemakers are cardiac implantable electronic devices (CIEDs) that deliver electrical impulses to regulate heart rhythm and manage conditions such as cardiac arrhythmias. While transvenous pacemakers (TP) have a generator with pacing leads, leadless pacemakers (LPs) are anchored within the right ventricular endocardium via tined or helical fixation. Device extractions may occur due to infection, dysfunction, or device upgrade.* *This study aims to analyze the incidence and severity of extraction complications between TPs and LPs.

A comprehensive narrative review was performed through the PubMed database. The following key search terms and synonyms were incorporated into the search algorithm: “transvenous pacemaker,” “leadless pacemaker,” “pacemaker extraction,” “device infection,” and “bacterial complications”. This yielded 788 entries. After uploading the search results, articles that focused on pacemaker extractions were included, while animal studies, pediatric studies, and studies that were not peer-reviewed were excluded. After excluding 691 studies, 97 original articles on pacemaker extractions underwent full-text analysis.

Both TP and LP extractions had high procedural success and low mortality rates. TPs were found to have procedural risks absent from leadless, namely, the development of arteriovenous fistula through subclavian access, tricuspid dysfunction, and atrial tearing. Examples of some differences between these studies include one LP study, which reported no change in mortality at a follow-up period of 18 months, and one TP study showed little change in mortality up to 24 months. Patients with TP and bacteremia have an increased risk of infection in the pocket and along the pacing leads. Infection of pacing leads has been shown to increase mortality and long-term complications. LPs are favored in many cases, for they minimize the incidence of these complications, have shorter extraction times, and have better recovery post-op in comparison to TPs.

LPs are associated with fewer extraction complications due to their smaller size and minimally invasive procedural approach, eliminating the use of a generator pocket and transvenous leads. Due to the incorporation of case studies, this review’s limited sample size may decrease external validity. Future studies should collect large-scale data to further analyze the extraction complications between TP and LP patients.

## Introduction and background

Cardiac implantable electronic devices in modern practice

Cardiac implantable electronic devices (CIEDs) have become a core aspect in the management of cardiac arrhythmias. Pacemakers, in particular, are widely used to regulate abnormal heart rhythms by delivering electrical impulses when the heart’s intrinsic conduction system is unable to maintain an appropriate rate. Their ability to prevent symptoms such as dizziness, syncope, and fatigue while reducing the risk of more serious complications has made them an essential aspect of modern cardiovascular care. As technology continues to advance and indications for implantation broaden, the use of CIEDs has increased significantly in clinical practice.

This rapid growth in device implantation has been accompanied by a rise in the need for device-related interventions, including lead revisions and extraction procedures. While CIEDs provide significant clinical benefits, their increased utilization has also introduced new challenges, particularly in the management of complications. Among these, device-related infections have emerged as a major concern. The incidence of CIED infections has risen in recent decades, leading to more frequent encounters with complex presentations such as large vegetations on device leads [1].

Types of pacemaker systems

Permanent pacemakers (PPM) are a type of CIED designed to maintain an adequate heart rhythm in patients facing cardiac arrhythmias. These devices are commonly indicated for patients with symptomatic bradycardia stemming from sinus node dysfunction, atrioventricular block, heart failure, and other causes. There are two types of PPM, namely TP and LP [2,3].

TPs are multi-component systems consisting of a subcutaneous pulse generator and one or more TP leads. The pulse generator is implanted subcutaneously along the upper chest and is connected to dedicated pacing leads via the subclavian or axillary veins. These leads have varied intracardiac implantation sites, most commonly within the right atrium or right ventricle. Depending on the reason for PPM implantation, patients may require single-chamber, dual-chamber, or cardiac resynchronization (triple-chamber) pacing. During device maintenance of the pocket generator, transvenous leads do not require full replacement if intact, and can be reused [2,3].

LPs, on the other hand, are self-contained, fully intracardiac devices that do not require a subcutaneous generator pocket or transvenous leads. LPs are delivered percutaneously, most commonly through the femoral vein, and are deployed directly into the right ventricle. These are then embedded within the endocardium to provide direct pacing. Modern LPs were designed with extraction in mind, with new designs featuring a docking interface, allowing for retrieval if necessary [2,3]. Common TP and LP structures and comparisons are outlined below in Table 1.

**Table 1:** Transvenous and leadless pacemaker structure and implantation comparisons *Implantation Locations: The location of implantation depends on the type of pacemaker. Single-chamber pacing will require either RA or RV implantation. Dual-chamber pacing will require RA and RV pacing. Triple-chamber pacing will require RA, RV, and LV pacing. The characteristics of the two different types of pacemakers are derived from the articles that discuss the key differences between TP and LP devices [2,4,5]. RA: right atrial; RV: right ventricular; LV: left ventricular; CRT: cardiac resynchronization therapy; TP: transvenous pacemakers; LP: leadless pacemakers

Pacemaker Type	Transvenous Pacemaker	Leadless Pacemaker
Pocket generator	Yes	None
Number of leads	1-3	None
Implantation locations*	RA, RV, and/or LV	RV
Implantation approach	Pocket creation + venous access	Venous access
Extraction features	Leads can be reused if intact	Docking/retrieval feature
Chamber / pacing modes	Single, dual, and CRT	Single only
Invasiveness	Moderate	Minimal

Indications for pacemaker extraction

Pacemaker extractions can occur due to device-related infection, malfunction, or upgrade. Device-related infections are associated with morbidity and mortality [6]. One cause of infection is the activation of the immune system by lead, which leads to fibrotic encapsulation [7]. This induces the formation of vegetation, which can obstruct the pulmonary arteries and increase the risk of mortality [1]. Thus, extraction of the pacemaker is required. Delay in diagnosis can worsen the disease and require advanced techniques for extraction [1,7]. Namely, if the vegetation grows larger than 2.5 centimeters, then an open surgery approach is recommended [1]. However, many patients do not qualify for these treatments due to their comorbidities [1].

Bacteremia is another indication for extraction, where *Staphylococcus aureus*,* P. aeruginosa*,* and S. marcescens* adhere to the CIED [8]. One study found that the incidence of CIED infection is from 0.5% to 2.2% [8]. The primary risk factors for CIED infections include the male sex, implantable cardioverter defibrillator leads, abandoned leads, and prior CIED procedures [9]. Chronic diseases can also increase the risk of infection [8]. Namely, diabetes can increase the risks of pocket infection complicated by infective endocarditis [9]. Besides patient-related factors, device- or procedure-related factors, like abrasion or skin erosion, also increase the risks of infection [9,10]. The standard treatment involves pacemaker extraction followed by IV antibiotics to fully eliminate bacteria [8]. In addition to infections, lead malfunction or lead fracture is another indication. One study that involved the removal of 1227 leads found lead dysfunction to be the most frequent indication [11-16]. These different indications for extractions can present with an array of complications unique to each type of pacemaker system. Thus, the two types of pacemakers should be fully examined to understand their differences in extraction risks.

Study rationale and objective

Although transvenous lead extraction is widely studied, existing research on extraction-related complications varies significantly amongst device types and the clinical situation, whether differing in procedural complexity or patient medical history. Specifically, there is limited consolidated data directly comparing complications between TP systems and newer models of LP systems, despite the distinct approaches both device designs and extraction methods consist of. This introduces the difficulty in being able to fully and effectively assess procedural risk, limiting the ability to guide a clinical decision when selecting a device. This study ultimately aims to systematically compare the incidence, severity, and nature of extraction-related complications between TP and LP systems in order to categorize the differences in risk profiles to efficiently guide patient-specific treatment strategies.

## Review

Methods

This study conducted a narrative literature review with a structured search strategy. Literature was collected on PubMed/MEDLINE through keyword-based searches. Namely, the following keywords were determined to be relevant to this study’s topic: “transvenous pacemaker,” “leadless pacemaker,” “pacemaker extraction,” “device infection,” and “bacterial complications.” To yield a comprehensive set of research studies, each reviewer used a different combination of keywords alongside the AND/OR boolean operators, which led to refined search results that include new sets of articles. This is appropriately displayed in Table 2.

**Table 2:** Methodology of search algorithm used for identifying articles to be used in the review article.

Sub-Topics	Search Algorithms
Transvenous Pacemakers	("Transvenous Pacemaker") AND (("extraction" OR ("antibiotic" OR ("infection severity") OR ("recurrence"))))
Leadless Pacemakers	(("Leadless pacemaker") AND (extraction) AND (mortality OR complications OR "patient outcome"))
Bacterial Infection	((Transvenous pacemaker) OR (leadless pacemaker)) AND ((Bacterial Infections) OR (Bacterial Disease) OR (Bacterial Diseases) OR (Bacterial Infection) OR (Infection, Bacterial) OR (Infections, Bacterial))
Extraction	(("Transvenous pacemaker") OR ("leadless pacemaker")) AND (("transvenous extraction") OR ("percutaneous extraction"))

**Figure 1:**
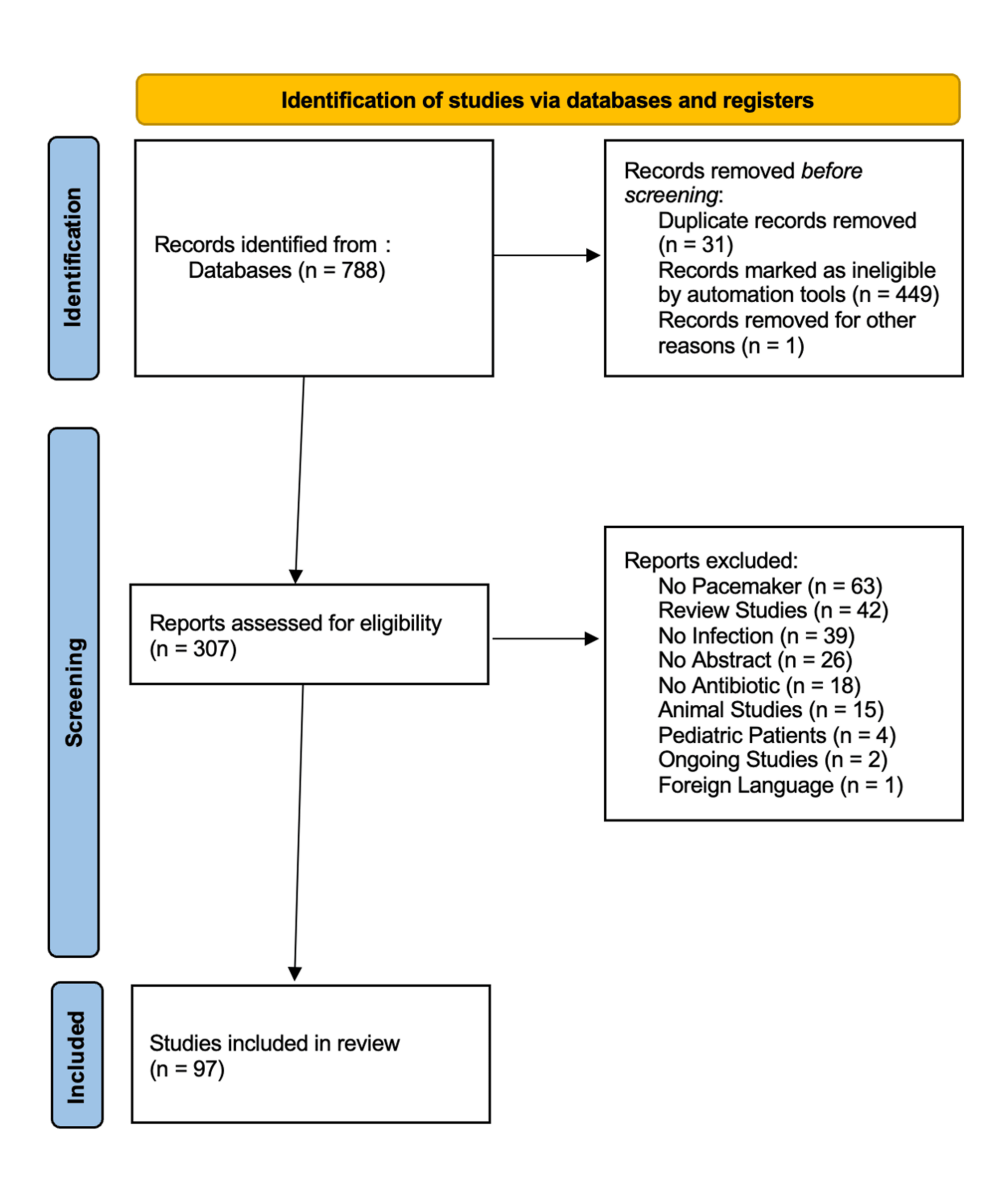
Methodology of the screening process used and articles included in the study This flow diagram shows the process of excluding irrelevant PubMed studies based on exclusion criteria, where the final number of included articles is 97.

The search strategy initially yielded 788 articles. Using PubMed’s automation tool, the results were restricted to studies that were published on or after 2015, with the exception of articles by Cho et al., Ruttmann et al., and Chang et al., which, upon agreement of the reviewers, were included due to them being case reports or case series that, other than the inclusion date, fit the inclusion criteria of this study [16-18]. This led to the removal of 449 articles. A total of 31 duplicate records were removed, while one unretrievable record was also excluded. From there, animal studies, pediatric studies, and non-peer-reviewed studies were excluded. Studies that examine CIEDs in general without specifying pacemaker-related results were also excluded. The included studies must meet the following inclusion criteria: participants must be adult patients who have undergone extraction of transvenous or leadless pacemakers. Another inclusion criterion is that studies must clearly report the extraction outcomes or complications. The process of screening is outlined in Figure 1.

For each study, the types of extracted pacemakers are classified as transvenous, leadless, or both. The method of extraction and extraction-related complications were also recorded as qualitative data. Afterwards, a qualitative narrative synthesis with selected illustrative studies was performed, which appropriately addressed the question this article is reviewing. Although most studies with quantitative data were observational, they can help illustrate associations between pacemaker types and treatment outcomes without raising ethical concerns. Case studies and case series were analyzed individually, which characterized complications associated with pacemaker extractions. Due to this being a narrative review, a formal quality assessment was not indicated.

Results

Study Characteristics

The majority of included studies utilized observational and descriptive designs. This includes case reports, case series, retrospective analyses, and single-center prospective studies. Sample sizes varied across studies, with most involving small cohorts through case report and case series designs, often having only a few patients. Studies opting for retrospective, single-center, or prospective methodologies often included larger samples than descriptive designs, having upwards of a few hundred patient samples. Follow-up duration was inconsistently reported across studies, with some not mentioning a follow-up interval. Among those that did specify a follow-up, many were relatively short, often at three months or less. A small number of studies extended beyond this, specifically those examining longer-term outcomes, such as mortality.

Procedural Success and Mortality

Procedural success rates were high for both transvenous and leadless pacemaker extractions. Studies reporting complete or clinically complete extraction outcomes for transvenous systems generally exceeded 90% [17-22]. Similarly, studies evaluating leadless pacemaker outcomes reported success rates above 90% in most cases [22-24]. Intraoperative mortality for TPs was exceptionally low, with most studies reporting no deaths during the extraction itself. Studies containing follow-ups had low mortality in the short-term following extraction, with less than 10% of cases expiring 30-days post extraction [20,22]. Studies with follow-up periods of up to 24 months show little change in mortality [18]. Likewise, studies evaluating intraoperative and short-term mortality for leadless extractions were also exceptionally low, with most studies reporting no deaths during extraction or within a 30-day follow-up period [24]. Studies with longer follow-up periods also had promising results, reporting no change in mortality at a follow-up period of 18 months [25]. These complications found in the studies in this review are summarized in Table 3 below.

**Table 3:** Summary of the studies used in finding the complication and outcomes of pacer extractions. The summary of these studies refer to the articles referenced below [11-16,19]. TP: transvenous pacemakers; LP: leadless pacemakers

Author + Year	Device Type	Indication for Extraction	Complications	Outcome	Follow-up
Villegas et al., 2022 [12]	LP	Need for system upgrade, threshold elevation, and PM syndrome	Pseudoaneurysm formation	Two patients were discharged 24 hours after the procedure, while one had a complication	N/A
Neuzil et al., 2024 [13]	LP	Anterior wall myocardial infarction	N/A	Successful extraction	N/A
Tułecki et al., 2021 [11]	Transvenous (initial), but later had a combination of TP and LP (Micra AV, Medtronic, Minneapolis, MN)	Lead-related tricuspid regurgitation	Tricuspid regurgitation due to lead impingement and right ventricular dilation	Successful transvenous RV lead extraction and successful implantation of LP	2 weeks, and found recurring tricuspid regurgitation
Milman et al., 2020 [14]	TP	Pocket infection, bacteremia, endocarditis, sepsis	Lead malfunction, venous occlusion	High procedural success	3 years
Caiati et al., 2019 [15]	TP	Endocarditis	Acute heart failure, pulmonary embolism, and acute respiratory distress syndrome	Successful	Mean duration of 22 ± 12 months
Cho et al., 2014 [16]	TP	Lead endocarditis	No complications	Successful	One patient was relapse-free at a six-month clinical visit, and the second patient showed no relapse after a 5-year follow-up
Muhlestein et al., 2022 [19]	TP	Pocket infections	Pericardial effusion, pocket hematoma, and tricuspid regurgitation	Complete extraction success in 92.1% of patients	N/A

Extraction-Related Complications in Transvenous Pacemakers

Transvenous lead extraction is a crucial procedure because not only can it resolve device-related issues, but it can also introduce risks that jeopardize a patient’s safety. Despite technical advancements, many complications remain serious, with rates reported as high as 12.4%, including tricuspid valve regurgitation, atrial tearing, and pericardial effusion [14,15]. These can occur because of pacing leads causing buildup of vegetation or intracardiac mass around the surrounding cardiovascular tissues, such as the superior vena cava and the tricuspid valve [26]. Although progress has been made in extraction techniques, leading to overall lowered complication rates, injury within the tricuspid valve tends to be an issue since it can lead to regurgitation and cardiac dysfunction [11].

Beyond structural injuries, lead-associated endocarditis accounts for many of the morbidities amongst patients throughout their extractions. Unlike complications around the subcutaneous pocket, endocarditis is associated with infection around the pacemaker and requires complete removal of the device to prevent any constant recurrence in about 3.8% of cases [19]. Extractions that are performed for indications such as endocarditis are accompanied by higher complication rates due to the presence of vegetations and fibrotic thickening along the lead. These changes increase procedural complexity, challenges for complete device removal, and risks for embolization [15]. Most importantly, studies have shown that adverse effects are not only due to vegetation, but a multitude of factors, including damage to the tricuspid valve, septic emboli, and abscess-forming pneumonia [16].

Extraction-Related Complications in Leadless Pacemakers

Current research suggests that leadless pacemakers are associated with fewer extraction-related complications compared to transvenous systems, mainly due to the absence of transvenous leads and the subcutaneous pocket [27]. By removing the need for intravascular leads, these systems may reduce common risks, including tricuspid valve regurgitation, venous or atrial tearing, and procedural complexity [28]. Some studies indicate that these devices, being less invasive and requiring shorter procedures, could lead to a decrease in post-operative recovery burden with shorter hospital stays as few as three days, compared to patients undergoing transvenous lead extraction [29]. Additionally, while leadless pacemakers have shown promising potential in achieving high atrioventricular synchrony and stable atrial capture thresholds [29], they still present unique challenges that are different from traditional pacemakers. 

Despite the advantages, leadless pacemakers can face specific retrieval challenges in certain cases, especially when devices have been implanted long-term and the anchor fixation type is variable [12,30]. As the duration the pacemaker has been implanted increases, the device site can possibly be complicated by myocardial tissue buildup, which raises the complexity of the extraction and the potential risk of vascular injury [12]. Retrieval often requires femoral venous access and specialized snare-based techniques such as the two-snare extraction method, especially if the device is misoriented or migrating near the inferior vena cava or other tricuspid valve annular areas [29-31]. While leadless pacemakers do address many of the risks seen in transvenous leads, it is important to note that long-term extraction data are still greatly limited. This highlights that while leadless pacemakers do offer a different safety approach, extraction-related complications remain an area of concern for these devices. 

Discussion

Both transvenous and leadless pacemaker extractions demonstrate high procedural success and low overall mortality, supporting the general safety of device extraction when clinically indicated in post-transcatheter aortic valve replacement (TAVR) populations [31]. However, as mentioned in the introduction, meaningful differences in extraction-related complications emerge and warrant consideration during procedural planning, as well as the initial device selection. In summary, as mentioned previously, transvenous systems are associated with substantially higher structural and infectious complications, including vascular injury, valvular dysfunction, and infection propagation along the leads [13,15]. In contrast, leadless pacemakers exhibit fewer structural complications, shorter procedural times, and more favorable recovery [32]. Collectively, these findings suggest that extraction risk is not uniform across pacemaker systems and is influenced not only by procedural technique, but by the device design as well.

The observed differences in extraction-related complications are closely linked to their distinct positioning and long-term tissue interactions. Transvenous leads traverse the venous system and cross the tricuspid valve, where chronic endothelial contact and motion promote progressive fibrotic encapsulation along the lead and at fixation sites. Over time, these adhesions integrate the lead with vascular structure, such that extraction may cause disruption of fibrotic attachments, leading to venous tearing, valvular injury, and associated structural complications [6,13,18]. In addition, the continuity between a subcutaneous generator pocket and intravascular leads provides a surface along which bacteria can adhere to and propagate [15].

In contrast, leadless pacemakers are fully intracardiac and have no additional hardware such as a subcutaneous pocket or leads, resulting in a more localized attachment and reduced surface area for fibrotic adhesion or bacterial vegetation [33]. Devices often include a specific retrieval feature, allowing for simpler extraction that typically does not involve disrupting local vasculature. Together, these device-specific interactions offer a mechanistic explanation for the complication patterns between transvenous and leadless pacemaker extraction.

LP systems include the Medtronic (Minneapolis, MN) Micra platform (Micra VR, Micra AV, and Micra AV2) and the Abbott (Abbott Park, IL) Aveir platform (Aveir VR and Aveir DR). The Micra VR provides single-chamber ventricular pacing, while Micra AV and AV2 incorporate accelerometer-based atrioventricular synchrony. The Aveir VR is a single-chamber ventricular device, whereas the Aveir DR system enables dual-chamber leadless pacing via device-to-device communication. The earlier Nanostim system (St. Jude Medical/Abbott), which utilized an active fixation helix, has been withdrawn from clinical use due to device-related issues, including premature battery failure and docking mechanism concerns. These were all different systems that could be utilized in current medical practices and should be approached based on patient pathology with careful consideration to meet FDA-specific criteria and applicability of utilizing these systems [34].

Several limitations of the literature should be considered when interpreting these findings. First, the evidence base is composed primarily of observational and case studies, which limit causal inference and may introduce selection and reporting bias in complication characterization. Second, experience with leadless pacemaker extraction remains comparatively limited due to the more recent adoption of these systems, resulting in smaller sample sizes and short follow-ups relative to transvenous cohorts. Third, substantial heterogeneity exists in the definition and reporting of extraction-related complications across studies, complicating direct comparison of event rates and severity. Finally, Embase, Cochrane CENTRAL, and conference proceedings (HRS, EHRA, ESC) were not searched as databases for articles, and this may have missed European registry experience and late-breaking LP retrieval data.

Future research should focus on prospective, standardized evaluation of pacemaker extraction outcomes across device types. Large multicenter registries and longitudinal cohort studies with clear definitions of extraction-related complications would better allow for comparison of rates and severity between transvenous and leadless systems. In particular, larger extraction cohorts and extended follow-up durations are needed to determine observed structural and infectious risk differences over time.

## Conclusions

Pacemaker extraction is generally safe and effective across transvenous or leadless pacers, but is associated with distinct complications based on the design of the device. TPs were noted to have high structural and infectious complications during extraction, whereas current literature suggests fewer reported structural complications with leadless systems. Consideration of extraction complications and patient-specific factors may therefore support the use of LPs in populations at higher risk for infection or future device replacement only if aligned with appropriate and FDA-approved usage. An example would be a patient who needs cardiac resynchronization therapy or true dual-chamber pacing due to heart failure with reduced ejection fraction and left bundle branch block, who cannot receive an LP as a like-for-like substitute. So, clinical scenarios and patient-specific pathologies need to be acknowledged before a clinical decision is made. Overall, these findings underscore the importance of incorporating long-term extraction consideration into initial pacemaker selection and device management strategies.
